# Content and analysis of a knowledge translation activity for an elder abuse detection tool: a descriptive study

**DOI:** 10.1186/s12877-021-02402-8

**Published:** 2021-08-06

**Authors:** Mark J. Yaffe

**Affiliations:** 1 Department of Family Medicine, McGill University, 5858 Cote –des-Neiges Road, Montreal, Quebec H3S 1Z1 Canada; 2 Family Medicine Centre, St. Mary’s Hospital Center, Integrated University Centre for Health and Social Services of West Island of Montreal, 3830 Lacombe Avenue, Montreal, Quebec H3T 1M5 Canada

**Keywords:** Elder abuse, Mistreatment, Older adults, Screening, Research, Information exchange

## Abstract

**Background:**

Knowledge translation (KT) is challenging to carry out and assess. The content of a program developed to foster KT activities pertaining to the Elder Abuse Suspicion Index (EASI)©, a tool to help identify elder abuse, is described, along with reporting and analysis of some of its outcomes.

**Methods:**

Enquiries about the use of the EASI were encouraged through completion of a structured questionnaire available on an EASI website. These were submitted by email and guided individualized responses. Descriptive data collated anonymously from the questionnaires described in aggregate corresponders’ occupations, countries of work, information needs about the tool, and intent of use. The processes that generated this data were evaluated as to whether they conformed to established elements of KT.

**Results:**

One hundred thirty-eight queries were received over 6 years coming from enquirers with 12 different professional backgrounds, working in 25 countries. The information sought aimed to facilitate EASI use in clinical, quality improvement, public health, research, teaching, KT, and commercial ventures.

**Conclusions:**

This activity, incorporating recognized elements of a KT undertaking, documents specific global interests in elder abuse detection. It suggests a model for researchers to gauge interest in their findings and to promote exchange around them.

**Supplementary Information:**

The online version contains supplementary material available at 10.1186/s12877-021-02402-8.

## Introduction

Mistreatment of older adults is not only a social issue, but also a cause of their premature morbidity and mortality [1] and increased utilization of hospital emergency and in-patient services [2]. Understanding the complexity of elder abuse manifestations, detection, management, and possible prevention appears to increasingly depend on translational research. The latter aims to ensure that knowledge generated by research reaches the people or populations intended, and then applied appropriately [3]. Such translational research requires application of implementation science, “the scientific study of methods to promote the systematic uptake of research findings and other evidence based practices into routine practice.” [4]. When principles of knowledge translation are applied to an entire research activity by having researchers and knowledge users as equal partners (with an aim of making the outcomes more relevant), the process has been described as integrated knowledge translation [5]. The specific optic used in this paper for elder abuse knowledge translation (KT) is that of “a dynamic and iterative process that includes synthesis, dissemination, exchange and ethically-sound application of knowledge to improve health, provide more effective health services and products, and strengthen the health care system” [6].

Elder abuse KT occurs globally through work of the International Network for Prevention of Elder Abuse [7], the United Nations supported World Elder Abuse Awareness Day [8], the World Health Organization [9], and through compilations that compare and contrast approaches to elder abuse and outcomes of interventions in diverse countries [10].

At a national level KT is seen in activities of government agencies such as the U.S. Centers for Disease Control and Prevention [11] and non-governmental organizations such as the Canadian Network for Prevention of Elder Abuse [12] and the National Center on Elder Abuse in the U.S. [13]. National forums have been used for exchange of expert opinion on elder abuse. In the U.S. the 2013 Forum on Global Violence Prevention of the Institute of Medicine and National Research Council of the National Academies [14] and the 2015 National Institute on Aging Conference on Undertsanding and Preventing Elder Abuse [15] are examples of this.

Regionally, state, provincial, municipal and grass-roots organizations also play important roles in educating about elder abuse prevention, identification and management, and in mobilizing relevant stakeholders (e.g. the public, clinicians, legislators). In Canada the government of the province of Quebec funds a unique Research Chair in Mistreatment of Older Adults, with the incumbent mandated for heavy engagement in elder abuse KT activities [16].

An appreciation of the potential for Knowledge Translation of elder abuse research can be assessed by consideration of the volume of work actually published. In 2007 Erlingsson’s systematic review of research papers on any aspect of elder mistreatment in three major databases between 1981 and the end of 2005 found 398 publications (an average of 15.9 per year) [17]. In 2011 Daly et al. published another systematic review of elder abuse research articles listed between 1975 and the end of 2008 using a larger number of data bases, and identified 590 papers (an average of 17.8 per year) [18]. In 2020 a scoping review by Burnes et al. of research on outcomes in elder abuse interventions appearing from 1986 to 2019 found 52 studies meeting broad inclusion criteria for this critical aspect of elder abuse work [19]. One observes therefore a growing body of research from which KT might arise.

Within the research community a common means for assessing KT and extent of impact of specifc works is use of citation indices which identify and record the number of times a particular work is referred to in another publication [20]. Given that such an approach has limitations, the objective of this paper was to explore, using a specific research-generated elder abuse detection tool as the subject of interest, whether other KT activities could be developed that not only promote exchange and information sharing, but also inform about who is interested in the subject and for what purpose.

## Method

In 2008 we published development and validation work on the Elder Abuse Suspicion Index (EASI)©, a tool intended for use in ambulatory family practice to raise suspicion about the presence of elder mistreatment to a degree sufficient to justify referral to social or adult protective services for more in-depth assessment [21]. In 2014, in response to high volume email enquiry about the tool, we started directing queries about it to an EASI© website [22] that included information about the EASI©, its adaptations (EASI-sa, for self-administration; EASI-ltc©, for use in long-term care institutions; and EASI-leo, for law enforcement officers in the field), and a growing number of linguistic versions (currently 15: Chinese, English, Estonian, Finnish, French, German, Hebrew, Japanese, Italian, Latvian, Nepali, Portuguese, Romanian, Spanish, Turkish).

Those wishing to make an enquiry about a specific way in which they hoped to use the EASI© were requested to complete and return by email a short English language questionnaire (Additional file 1) found on the website. It was created by members of our research team in a series of brain-storming sessions aimed at identifying possible reasons, situations, and ways for using / administering the EASI. We also identified factors that might enable us to contextualize the enquiries since each one was to receive an individualized response. This questionnaire was designed to have predominantly tick-off response options to make completion time short, as well as to facilitate those whose primary language was not English.

The questionnaire therefore asked for names of the enquirers, institutions worked for, and postal and email addresses. Questions (with Yes/No/ Not Applicable response options and space for elaboration) recorded enquirers’ desired needs for the EASI©: clinical site (community agency, office, clinic, hospital); context of care (for profit, public, government); research (career research, student degree requirement); academic (teaching, student course work, article writing for journal, book chapter); public presentations: (conference, community); accreditation requirement; need for language translation or specific word change; format of use (paper, electronic health record); and commercial (for profit). This structured approached facilitated responses by a designated team member usually within a maximum of 7 days.

We hypothesized that a descriptive analysis of data from the questionanires would provide useful information about who was interested in KT around detection of elder abuse, and for what reasons. These data were therefore collated in anonymized aggregate form for the 6 year period of July 2014 to June 2020. This paper addresses what was found in the subsequent analysis, as well as implications for Knowledge Translation in the field of Elder Abuse.

## Results

One hundred thirty-eight questionnaires making enquiry about use of the EASI tool were received over the 6 year period. As listed alphabetically in Table 1, they came from at least 25 countries (for 5 enquiries the country was not legible or absent). The largest number of queries originated in the United States: a total of 71, from 28 /50 states (ranging from 1 to 8 queries per state). The next largest came from Canada: 16 in total, from 5 /10 provinces (ranging from 1 to 7 queries per province).

**Table 1:** Provenance of enquiry

Australia	
Bahrain	
Belgium	
Canada	
China	
Estonia	
Greece	
Hong Kong	
India	
Indonesia	
Iran	
Ireland	
Jordan	
Latvia	
Mongolia	
Nepal	
New Zealand	
Pakistan	
Philippines	
Portugal	
Saudi Arabia	
Spain	
Sweden	
Turkey	
United States	

The occupational backgrounds of the enquirers are summarized in Table 2. A quarter were physicians from a broad spectrum of disciplines, while slightly less than a quarter came from nurses. The remainder, in descending order, came from social workers (17.2%), psychotherapists of varying backgrounds (11.8%), representatives of commercial ventures (10.2%), and a small percentage of other backgrounds.

**Table 2:** Background of enquirers

Enquirer Background	Number^a^	Percent
Physician (Internist, Family Physician / General Practitioner, Emergentologist, Geriatrician, General Surgeon, Orthopedic Surgeon; Public Health Officer)	32	25.0%
Nurse or Nurse Practitioner	29	22.7%
Social Worker	22	17.2%
Psychotherapist (psychologist, counsellor, behavior scientist)	15	11.8%
Commercial (electronic medical record / informatics company; Intellectual property; Book company; survey company)	13	10.2%
Researcher (sociologist, methodologist, public health officer, economist)	8	6.3%
Rehabilitation (physical therapist, occupational therapist)	3	2.3%
Legal / advocacy services	2	1.6%
Audiologist	1	0.8%
Para-Medic	1	0.8%
Long Term Care Organization	1	0.8%
Dentist	1	0.8%
Total	128	100%

^a^ 10 missing values

As reported in Table 3, 136/138 enquirers listed one or more reasons for requesting use of the EASI (2 were not identifiable). 42.6% (58/136) collectively fell within our classification of “clinical” activities, compared to 30.9% (42/136) which were “academic” in nature. 18.4% (25/136) could be allocated to either of those two groups, and were assigned to a third category, “clinical or academic”. The remaining 9.6% (13/136) of correspondence was of a commercial nature coming from ventures with potential copy-right concerns.

**Table 3:** Projected EASI© uses

Planned EASI use	Number^a^	%	Context of Use
Clinical practice	51	37.5%	Clinical
Academic Research project	27	19.9%	Academic
Language Translation of tool	13	9.6%	Clinical or Academic
University degree requirement (research or teaching project)	13	9.6%	Academic
Copy-right / Commercial: (electronic medical record / informatics companies; Intellectual property; Book company; survey company)	13	9.6%	Commercial
Article in journal or professional publication	6	4.4%	Clinical or Academic
Article in book chapter	6	4.4%	Clinical or Academic
Project of Public Health, Quality Improvement or Long-term care facility	5	3.7%	Clinical
Conference Presentation	2	1.5%	Academic
Advocacy / Legal activities	2	1.5%	Clinical
Total	136	100.0%	

^a^ 2 were not definable

## Discussion

This paper examined a specific KT project around an elder mistreatment detection tool, the Elder Abuse Suspicion Index (EASI)©. The outcomes of that activity can be examined within a framework of recognized elements of KT, and provide a snapshot of interest in elder abuse, by whom, and for what purpose.

### Synthesis

One element of Knowledge Translation is Synthesis, which includes the “… integration of research findings … .within a larger body of knowledge on the topic” [6]. Since the EASI© website was not constructed to record “hits”, we do not have information on the total number of times the site was consulted. However a sub-sample of that total has been examined in this study, involving individuals from varied backgrounds and locations who corresponded to express intent to actively engage in a broad range of synthesis activities. Just under two thirds of them worked in the U.S. and Canada, possibly the reality of those countries larger populations compared to the majority of other countries from which correspondence originated. It is noteworthy that from those two countries enquiries came from a broad geographical distribution, suggesting that synthesis was not limited to a particular region. This may reflect the influence of their diverse governmental and non-governmental organizations, or perhaps to particular attention paid within those jurisdictions to copy-right issues.

### Dissemination

A second element of Knowledge Translation is Dissemination [6]. Table 3 suggests that just over 50% of the enquiries had potential clinical application, and these came from a large number of different clinical professionals. Noteworthy is that while the EASI was developed and validated for use by family physicians in the ambulatory setting, family doctors/general practitioners comprised only one of eight different types of physicians making enquiry. Further, since 75% of all queries came from non-physicians of a multidisciplinary nature it would appear that KT was occurring outside the original target population for the tool. Of note, social workers ranked third, below physicians and nurse in the percentage of enquiries. Since this profession has had longstanding involvement in elder abuse detection, intervention, victim protection and advocacy [23], it is possible that the EASI is of less interest to them.

Table 3 shows that within the academic community there was a focus on dissemination, an expected academic role: just under 50% of enquiries appeared motivated by plans for journal article and book chapter writing, as well as by the giving of community and conference presentations, teaching of students and engaging in research.

Interestingly, while the original EASI research publication and website appeared in English, as did the website and its questionnaire, 4/5 of the countries from which queries originated had a language other than English as their primary language. This might suggest that, at least for the sample studied, language was not a large obstacle to information sharing. We hypothesize, in fact, that the presence of our structured questionnaire may have facilitated enquiry from those who otherwise might have had difficulty corresponding in a language other than their own.

### Exchange

Exchange, a third element of Knowledge Translation, is the “linkage and knowledge exchange between researchers and knowledge users” [6]. While not evident from the results reported in this paper, the questionnaire content and format was likely a factor that facilitated a rapid, personal response to each enquiry. At times this activity expanded into what might be considered as spontaneous brief mentorship supplying advice about EASI use, its strengths and limitations, who could administer the tool, and how results might be interpreted and ethically applied. This approach also sometimes generated broader networking through invitations for the EASI team for conference presentations and research collaborations.

Another important linkage between researchers and knowledge users occurred with the creation of thirteen new linguistic versions of the EASI (beyond the English and French versions validated by our team), the requests for such translations initiated by individuals or teams world-wide. They sought varying degrees of collaboration that included us reviewing back- translations into English of the various new linguistic versions in order to ensure fidelity to the original English language tool. The result was often stimulating email exchanges discussing word nuances in different countries. In a few cases we were able to recall individuals who had common interest to translate into a particular language, and after email enquiry as to whether they might want to be linked up, such connections were facilitated by us.

### Ethical application of knowledge

A fourth element of KT is the expectation to “use ethically – sound application of knowledge” [6]. While examples of this are not directly evident from results in this paper, the EASI© website does provide evidence for such an ethical approach: the authors of new linguistic versions of the tool were extended the opportunity to have their translations posted on the website, credited to their names and professional affiliations. In this way their interest in elder abuse might receive greater exposure, while communities other than their own might have access to their linguistic contributions.

### Limitations

While the methodology used in this KT activity is likely applicable to other subjects, the descriptive results are not generalizable. They are derived from individuals who found the EASI website on their own (and hence the enquiry questionnaire), or who were directed to it following email correspondence to our team. This is acceptable however since the goal of this work was to describe development and implementation of an elder abuse KT activity. Outcomes of KT do not necessarily have to be generalizable; rather they are a reflection of a specific sample who engage in KT within a particular time frame.

## Conclusions

Knowledge Translation is a common goal for both researchers and their funders; however it is complex to define, carry out, and demonstrate impact. This paper has shown, using a product of elder abuse research as an example, that it is possible to create practical activities that meet elements expected of a KT undertaking. The high number of enquiries from international and multidisciplinary sources with a broad range of stated needs suggests this KT activity has potential to positively impact on the well-being of older adults.

## Supplementary Information

**Additional file 1.**

## Data Availability

All data generated during this study has been included in this published article. However any enquiry about it may be requested from the corresponding author, at mark.yaffe@mcgill.ca.

## Abbreviations

KT: Knowledge Translation
CIHR: Canadian Institutes of Health Research

## References

[1] Lachs MS, Williams CS, O'Brien S, Pillemer KA, Charlson ME (1998). The mortality of elder mistreatment. JAMA.

[2] Yunus RM, Hairi NN, Choo WY (2019). Consequences of elder abuse and neglect: a systematic review of observational studies. Trauma Violence Abuse.

[3] Wolff SH (2008). The meaning of translational research and why it matters. JAMA.

[4] Eccles MP, Mittman BS (2006). Welcome to implantation science. Implementation Sci.

[5] Canadian Institutes of Health Research. Guide to knowledge translation. https://cihr-irsc.gc.ca/e/45321.html. Retrieved June 15, 2021.

[6] Canadian Institutes of Health Research. Knowledge translation. https://cihr-irsc.gc.ca/e/29418.html. Retrieved 15 Dec 2020.

[7] International Network for Prevention of Elder Abuse. www.inpea.net. Accessed 15 Dec 2020.

[8] World Elder Abuse Awareness Day. United Nations. www.un.org. Accessed 15 Dec 2020.

[9] World report on ageing and health (2015). World Health Organization.

[10] Phelan A (2013). International perspectives on elder abuse.

[11] Elder abuse surveillance: Uniform definitions and recommended core data elements (2016). Centers for Disease Control and Prevention. https://www.cdc.gov/violenceprevention/pdf/ea_book_revised_2016.pdf Accessed 15 Dec 2020.

[12] Canadian Network for the Prevention of Elder Abuse. www.cnpea.ca. Accessed 15 Dec 2020.

[13] National Center on Elder Abuse. https://ncea.ac.gov. Accessed 15 June 2021.

[14] IOM (Institute of Medicine) and NRC (National Reserch Council) (2014). Elder abuse and its prevention: workshop summary.

[15] National Institute on Aging. https://www.nia.nih.gov/research/dbsr/nih-workshop- multiple-approaches- understanding- and-preventing-elder-abuse. Accessed 17 June 2021.

[16] Quebec Chair on Elder Abuse. https://maltraitancedesaines.com/en/ . Accessed 17 June 2021.

[17] Erlingsson CL (2007). Searching for elder abuse: a systematic review of database citations. J Elder Abuse Negl.

[18] Daly JM, Merchant ML, Jogerst GJ (2011). Elder abuse research: a systematic review. J Elder Abuse Negl.

[19] Burnes D, MacNeil A, Nowaczynski A, Sheppard C, Trevors L, Lenton E, Lachs MS, Pillemer K (2020). A scoping review of outcomes in elder abuse intervention research: the current landscape and where to go next. Aggress Violent Behav.

[20] Measuring your impact: Impact Factor, citation analysis, and other metrics. University of Illinois Chicago University Library. https://researchguides.uic.edu/c.php?g=252299&p=1683205. Accessed 15 Dec 2020.

[21] Yaffe MJ, Wolfson C, Weiss D, Lithwick M (2008). Development and validation of a tool to assist physicians’ identification of elder abuse: the elder abuse suspicion index (EASI © ). J Elder Abuse Neglect.

[22] Elder Abuse Suspicion Index. https://www.mcgill.ca/familymed/research/projects/elder. Accessed 15 Dec 2020.

[23] Wolf R, Bonnie RJ, Wallace RB (2003). National Research Council. Elder abuse and neglect; history and concepts. Elder mistreatment: abuse, neglect, and exploitation inan aging America.

