# Supplementary material for: Content and analysis of a knowledge translation activity for an elder abuse detection tool: a descriptive study
Source: BMC Geriatr. 2021 Aug 6;21:455. doi: 10.1186/s12877-021-02402-8 (PMC8349046; doi:10.1186/s12877-021-02402-8)
Supplement: Supplementary file 1 — Additional file 1. [file 12877_2021_2402_MOESM1_ESM.rtf]

Appendix 1        Permission for EASI© use 
Thank you for your interest in the Elder Abuse Suspicion Index (EASI) ©. Since we receive a large number of different enquiries, we are trying to simplify and speed up the process of response. Kindly complete this questionnaire in its entirety, even if most answers are No or N/ A (non-applicable). 
Kindly scan the completed questionnaire and return it as a PDF email attachment to: mark.yaffe@mcgill.ca
1)	Your name and/ or the organization that you represent 

				
2) 	Email address: 				
3) 	
Postal address:
 				
4) 	Would your use of the EASI be for research purposes? 	Yes 	No 		
5) 	Describe the nature/ context of the research. 			N/A 	
6) 	Would your use of the EASI be for other academic activities? 	Yes 	No 		
7) 	Describe the nature/ context of this academic work, e.g. journal 			N/A 	
	manuscript, book chapter writing or book. 				


					
8) 	Do you need to translate the EASI into another language? If yes, which? 	Yes 	No 		
9) 	Is there a word or words that you feel might need to be changed in order      	Yes 	No 		
	to reflect usage in your community? If yes, indicate what?

 
cccc				
					
10) Would your use of the EASI be for clinical care? 	Yes 	No 		
11) Will your clinical use of the EASI be limited to yourself? 	Yes 	No 		
12) If clinical use involves others, approximately how many? 			N/A 	
13) Will your use of the EASI take place in an office setting? 	Yes 	No 	N/A 	
14) Is that office setting government- run or non-profit? 	Yes 	No 	N/A 	
15) Will your use of the EASI take place in a clinic setting? 	Yes 	No 	N/A 	
16) Is that clinic setting government- run or non-profit? 	Yes 	No 	N/A 	
17) Will your use of the EASI take place in a hospital setting? 	Yes 	No 	N/A 	
18) Is that hospital setting government- run or non-profit? 	Yes 	No 	N/A 	
19) Will your use of the EASI take place in a community setting? 	Yes 	No 	N/A 	
20) Is that community setting government- run or non-profit? 	Yes 	No 	N/A 	
21) Is your interest in the EASI linked to accreditation requirements? 	Yes 	No 	N/A 	


22) Will you use the EASI in a paper format? 	Yes 	No 	N/A 	
23) Will you use the EASI in an Electronic Health Record 	Yes 	No 	N/A 	
24) f you will use the EASI in an Electronic Health Record, does its software 	Yes 	No 	N/A 	
belong uniquely to you? 				
25) If you will use the EASI in an Electronic Health Record, is its 	Yes 	No 	N/A 	
commercial, i.e. one that has propriety ownership? 				
26) Would your use of the EASI have commercial/ profit implications for 	Yes 	No 	N/A 	
you? E.g.as a text book publisher or a creator/ distributor of 				
proprietary Electronic Health Records? 				
